# Cardiovascular Disease (CVD) Risk Assessment Among University Employees in India and Its Implications on Planning Worksite CVD Prevention and Health Promotion Interventions

**DOI:** 10.7759/cureus.86642

**Published:** 2025-06-24

**Authors:** Abhijeet H Jagtap, Uma S Mahajan, Abhay M Kudale

**Affiliations:** 1 Public Health, Punyashlok Ahilyadevi Holkar Solapur University, Solapur, IND; 2 Epidemiology and Public Health, School of Health Sciences, Savitribai Phule Pune University, Pune, IND; 3 Biostatistics, School of Health Sciences, Savitribai Phule Pune University, Pune, IND

**Keywords:** cvd risk assessment, india, prevention and health promotion worksite interventions, risk factors, university employees

## Abstract

Introduction: University employees worldwide have a relatively higher prevalence of cardiovascular disease (CVD) risk factors. However, in the Indian context, most University employees are not regularly screened for CVD risk factors. Against this background, a cross-sectional study was conducted to determine the prevalence of hypertension and CVD risk factors among Punyashlok Ahilyadevi Holkar Solapur University employees in India.

Material and methods: This cross-sectional study included the university's teaching, administrative, and support staff (n=126). Data on physiological, anthropometric, worksite, and sociodemographic variables were collected using the WHO Stepwise approach to noncommunicable disease (NCD) risk factor surveillance (STEPS) questionnaire. The chi-square/Fisher's exact test was used to test the association of employees' sociodemographics, work-related addictions, diet intake, exercise, and health-related characteristics with CVD risk. The univariate and multivariable logistic regression analysis assessed the factors associated with CVD risk.

Results: The prevalence of hypertension was found to be 42%. There was a clustering of NCD risk factors (≥ 3 risk factors) in 88% (n=111) of employees. Overweight and obesity were the most prominent risk factors identified in 64% (n=81) of the employees, followed by family history of CVDs (62%, n=78), unhealthy dietary habits (61%, n=77), abdominal obesity (45%, n=57), and raised blood pressure (42%, n=53). Further, men's gender, age of more than 40 years, family structure, consumption of alcohol, smoking, higher monthly income, and academic and regular employment were the risk factors significantly associated with future CVD development risk.

Conclusion: University employees are prone to developing CVDs, and urgent attention is needed to develop worksite CVD prevention and health promotion interventions.

## Introduction

The cardiovascular disease (CVD) risk burden in the Indian population is a multifaceted issue influenced by a combination of lifestyle, genetic, and socioeconomic factors. The prevalence of CVDs in India is rising, driven by rapid urbanization, dietary changes, and increased stress levels. This trend is further complicated by the interplay of mental health disorders, which exacerbate cardiovascular risks and complicate treatment outcomes. The burden of CVD risk factors is evident across various demographics, with significant variations observed between rural and urban populations, as well as among different socioeconomic groups [1,2]. Cardiovascular diseases are a leading cause of mortality in India, with significant geographic and sociodemographic variations in risk factors [3-7]. Smoking, diabetes, dyslipidemia, and hypertension are prevalent risk factors, with smoking being more common in rural areas, while other risk factors are more prevalent in urban settings. The risk of CVD is higher among older adults, with primary risk factors including hypertension, diabetes, dyslipidemia, obesity, and smoking. These factors are exacerbated by stress and genetic predisposition [4,5].

Screening for CVDs is a critical public health strategy aimed at early detection and prevention of these conditions. Screening for risk factors in CVD management offers numerous benefits, including early detection, risk stratification, and the implementation of preventive measures to significantly reduce CVD morbidity and mortality [3]. Integrating screening tools, such as the WHO CVD risk assessment charts and risk assessment strategies, into clinical practice can improve patient outcomes through timely interventions and personalised care plans. If cost-effective interventions are used, opportunistic screening for CVD risk factors can be widely applied in low- and middle-income countries. This approach can prevent and control CVD by identifying high-risk individuals and providing affordable treatment options [8].

A multitude of lifestyle, occupational, and demographic elements significantly affect the risk factors associated with CVD among university personnel. These factors include a lack of physical activity, suboptimal dietary habits, psychological distress, and a range of additional health-related behaviours. The incidence and ramifications of these risk factors exhibit considerable variation across distinct cohorts within the university environment, including faculty members, administrative personnel, and students. A comprehensive understanding of these risk factors is crucial for developing targeted interventions that enhance cardiovascular health among university employees [9-15].

The assessment of health risks among employees constitutes the initial phase in implementing worksite health promotion (WHP) and CVD prevention initiatives within any occupational environment. It is essential to identify the intrinsic and behavioural risk factors of employees to effectively plan, evaluate, and develop an appropriate worksite health promotion strategy [16-18].

The WHPs implemented by healthcare professionals within occupational settings can exert a significant influence. By addressing the modifiable risk factors present among the workforce, these initiatives have the potential to diminish the risk of premature mortality attributable to CVDs. To avert the onset of CVDs in the future, it is crucial to concentrate efforts on high-risk employees by identifying a range of CVD risk factors, with particular emphasis on psychosocial determinants. Given the heterogeneous working population in the Indian context, it is essential to conduct research across various sectors of employment to evaluate CVD risk and the viability of WHPs [19-21].

While the studies highlight the significant presence of CVD risk factors among university employees, they also underscore the importance of targeted interventions to address these risks. Implementing workplace wellness programs, promoting physical activity, and improving dietary habits can significantly reduce the prevalence of these risk factors. Regular health screenings and stress management programs can help enhance the cardiovascular health of university employees. Although the existing body of literature establishes a comparatively high prevalence of diverse non-communicable disease (NCD) risk factors among university personnel on a global scale [11-15], the phenomenon has not been extensively examined within the Indian context. A limited number of investigations have been conducted in various university environments in India, which have indicated the existence of numerous CVD risk factors such as hypertension, overweight and obesity, sedentary behaviour, and dietary practices among university staff [10,22,23].

According to the All-India Survey on Higher Education (AISHE) report released for the academic year 2021-2022, there are a total of 1,113 universities officially recognized in India, and the aggregate number of academic, non-teaching, and ancillary personnel employed across these institutions approximates 2.95 million [24]. This figure signifies a substantial demographic that has been inadequately explored in relation to the prevalence of CVD risk factors. Conducting screenings of university employees throughout India for CVD risk factors is crucial for identifying at-risk groups and for developing a population-based preventive strategy aimed at mitigating and managing CVDs. Workplace screenings facilitate the detection of previously undiagnosed hypertension and other risk factors, enabling early intervention and treatment that can diminish the incidence of premature mortality and morbidity associated with CVDs; concurrently, they aid in the strategic planning of CVD prevention and health promotion initiatives [18-20].

In light of this context, a cross-sectional study was executed at a state university in India to address this pressing need. The objectives of the study include determining the prevalence of CVD risk factors among university employees, evaluating the prospective risk of developing CVD within this population, and investigating the correlation between CVD risk and other documented variables.

## Materials and methods

This study is a cross-sectional investigation conducted at the university health centre between June and August 2022. Ethical clearance for the study was obtained from the Institutional Ethics Committee of Savitribai Phule Pune University (Pune, MH, IND) before the study (approval no. SPPU/IEC/2019/07). We obtained the list of all the employees from the university office. This list included all the teaching and non-teaching staff of the university. From the total number of employees (n=369), only those employees who were of age ≥ 40 years and who gave consent for participation in the study were included (n=126) in the survey. The age criteria for selecting study participants were based on the requirements of the study tool, the WHO non-lab-based CVD risk assessment chart, which necessitated the inclusion of only employees aged≥ 40 years [25].

A screening camp was organised for these employees at the study site. At the start of the screening camp, the purpose and schedule were communicated to employees well in advance via an office circular. Before attending the screening camp, employees were asked to refrain from smoking, drinking tea or coffee, or engaging in vigorous activity. Each participant was initially interviewed using a structured questionnaire based on the WHO Stepwise approach to noncommunicable disease (NCD) risk factor surveillance (STEPS) question-by-question (Q-by-Q) guide for NCD risk factor surveillance to gather data on basic sociodemographic characteristics and CVD risk factors [26]. These employees underwent a comprehensive clinical evaluation, which included anthropometric assessments involving body mass indices, waist and hip measurements, and blood pressure (BP) measurements. Blood pressure measurements were obtained and classified per the Indian hypertension guidelines-II [27]. The procedure for this entailed asking the employee to sit quietly for five minutes in the waiting room. Post which, the employee was sent for a BP measurement. The BP measurement technique was explained to the employee, and they were instructed not to talk or engage in conversation while their BP was being measured. Trained nursing staff measured the BP using the OMRON-HBP1300 (Omron Healthcare, Kyoto, JPN) digital BP apparatus as recommended by STRIDE-BP, an international scientific non-profit organisation founded by hypertension experts with the mission of improving the accuracy of BP measurement, the diagnosis, and management of hypertension.

Blood pressure measurements were taken with the employee sitting comfortably in a chair with arms fully bared and supported at the level of the heart, with feet resting on the floor. An appropriate BP cuff size was used to measure the BP levels. Three BP readings were taken at an interval of five minutes. The average of these three readings was calculated and recorded as the BP levels. An employee was labelled as normotensive or normal if their systolic BP (SBP)/diastolic BP (DBP) readings were between 130-139/85-89 mmHg. This cut-off was based on the guidelines given by the Indian Society of Hypertension [27]. Employees whose SBP/DBP levels were found to be higher than 140/90 mm Hg were called upon in subsequent weeks for follow-up checkups for three consecutive weeks. At subsequent visits, BP levels were again measured using a standardised approach. If, even on following visits, the SBP/DBP levels were found higher than 140/90 mm of Hg, the employee was classified as a new case of hypertension. Due to feasibility issues, we utilised office BP monitoring to classify employees as hypertensive or normal. Hypertensive employees were advised to seek professional medical help for the initiation of treatment and counselling.

Employees with higher BP readings were called upon for repeated measurements on subsequent days to confirm their hypertensive status. We used the WHO BMI classification, explicitly designed for the Asian adult demographic, to categorise the study participants. The CVD risk evaluation was derived from non-laboratory-based CVD risk charts developed by the WHO for application in resource-constrained environments. These non-laboratory-based CVD risk evaluation charts are formulated to estimate a decade of prospective CVD risk based on body mass index, SBP values, and smoking history in individuals aged 40 years or older and have been demonstrated to be effective within the Indian demographic [8,25,27].

The continuous variables were described as median and interquartile range (IQR), and categorical variables as frequencies (n) and percentages (%). The chi-square/Fisher’s exact test, Mann-Whitney test, and Z statistics were used to test the association of employees’ sociodemographics, work-related addictions, diet intake, exercise, and health-related characteristics with CVD risk. The univariate and multivariable logistic regression analysis assessed the factors associated with CVD risk. Initially, all the independent variables were entered in the multivariable logistic regression model, and the variables were removed using a backward stepwise elimination approach. The likelihood ratio test was used to compare the models at each step, and the variables with a p-value ≤0.10 were kept in the final model. The final multivariable model was adjusted for age, gender, and pre-existing NCDs. The 95% confidence interval (CI) was calculated for CVD risk and associated factors. The statistical analysis was done using Stata 12.1 (StataCorp LP, College Station, TX, USA).

## Results

The sociodemographic and other employee profiles are summarised in Table 1. There was a clustering of NCD risk factors (≥ 3 risk factors) in 88% (n=111) of employees (Table 2). Overweight/obesity was the most prominent risk factor identified in 64% of employees (n=81), followed by family history of CVDs (62%, n=78), unhealthy dietary habits (61%, n=77), abdominal obesity (45%, n=57), and raised BP (42%, n=53) (Table 3). Out of 126 university employees, the CVD risk was low in 53% of employees (n=67; 95% CI=44.46-61.89), and it was mild/moderate in 47% of employees (n=59; 95% CI=38.1-55.54). Around 18% of employees (n=14) were found to have a 10-year risk of developing CVDs in the future. The CVD risk showed a significant positive correlation (r(126) = 0.288, p <0.001) with the number of risk factors present in the individual. The CVD risk was significantly mild/moderate among the employees aged 52 years (IQR=46- 57) (p <0.0001), male (p=0.038), having nuclear families (p=0.085), who were head of the family (p=0.011). The CVD risk was significantly mild/moderate among regular employees (p <0.0001), employees working in academics (p=0.026), who had worked more than 10 years in the university (p=0.032), and whose monthly income was >30,000 Indian rupees (INR) (p=0.018).

**Table 1:** Association of sociodemographic and work-related factors with CVD risk among university employees Descriptive statistics for categorical variables are shown as n (%) and for continuous variables as median (IQR). The p-values are calculated using ^#^Fisher’s exact test, ^$^Mann-Whitney U test, and the chi-square test. *p-value significant at p ≤ 0.05, **p-value significant at p ≤ 0.01 IQR: Interquartile range, CVD: Cardiovascular disease, INR: Indian rupees

Variables	CVD risk					Chi-square/	
	Low; n (%)	Mild/moderate; n (%)		Total; n (%)			
						z statistics	p-value
Total employees	67 (53.2)	59 (46.8)		126			
Sociodemographic							
Age (years)							
Median (IQR)^$^	44 (41, 47)	52 (46, 57)		46 (42, 52)		-5.833	<0.0001**
Gender						4.283	0.038
Female	13 (76.5%)	4 (23.5%)		17 (13.5%)			
Male	54 (49.5%)	55 (50.5%)		109 (86.5%)			
Marital status						1.145	0.468^#^
Unmarried	0 (0.0%)	1 (100.0%)		1 (0.8%)			
Married	67 (53.6%)	58 (46.4%)		125 (99.2%)			
Type of family						2.972	0.085
Joint	28 (63.6%)	16 (36.4%)		44 (34.9%)			
Nuclear	39 (47.6%)	43 (52.4%)		82 (65.1%)			
Total household members						5.142	0.023*
≤4	32 (44.4%)	40 (55.6%)		72 (57.1%)			
>4	35 (64.8%)	19 (35.2%)		54 (42.9%)			
Relationship with the family head						6.485	0.011*
Self	42 (46.2%)	49 (53.8%)		91 (72.2%)			
Other	25 (71.4%)	10 (28.6%)		35 (27.8%)			
Education						0.511	0.475
Below graduate	16 (59.3%)	11 (40.7%)		27 (21.4%)			
Graduate & above	51 (51.5%)	48 (48.5%)		99 (78.6%)			
Religion						0.407	0.523
Non-Hindu	8 (61.5%)	5 (38.5%)		13 (10.3%)			
Hindu	59 (52.2%)	54 (47.8%)		113 (89.7%)			
Social category						6.092	0.014*
Reserved	42 (63.6%)	24 (36.4%)		66 (52.4%)			
Open	25 (41.7%)	35 (58.3%)		60 (47.6%)			
Work-related							
Department type						4.954	0.026*
Non-academic	46 (61.3%)		29 (38.7%)		75 (59.5%)		
Academic	21 (41.2%)		30 (58.8%)		51 (40.5%)		
Monthly income (INR)						5.579	0.018*
≤30,000/-	28 (68.3%)		13 (31.7%)		41 (32.5%)		
>30,000/-	39 (45.9%)		46 (54.1%)		85 (67.5%)		
Nature of service						13.579	<0.0001**
Contract	26 (81.3%)		6 (18.8%)		32 (25.4%)		
Regular	41 (43.6%)		53 (56.4%)		94 (74.6%)		
Cadre						0.412	0.521
Non-teaching	50 (54.9%)		41 (45.1%)		91 (72.2%)		
Teaching	17 (48.6%)		18 (51.4%)		35 (27.8%)		
Class						0.959	0.327
Class 1 & 2	26 (48.1%)		28 (51.9%)		54 (42.9%)		
Class 3 & 4	41 (56.9%)		31 (43.1%)		72 (57.1%)		
Nature of work						0.861	0.650
Professional	19 (48.7%)		20 (51.3%)		39 (31.0%)		
Office	23 (59.0%)		16 (41.0%)		39 (31.0%)		
Skilled/other	25 (52.1%)		23 (47.9%)		48 (38.1%)		
Service experience (years)						4.614	0.032*
≤10 years	24 (68.6%)		11 (31.4%)		35 (27.8%)		
>10 years	43 (47.3%)		48 (52.7%)		91 (72.2%)		
Median (IQR)^$^	12 (9, 15)		23 (13, 30)		14 (10, 23)	-5.321	<0.0001**

**Table 2:** Association of addictions and diet intake factors with CVD risk among university employees Descriptive statistics for categorical variables are shown as n (%) and for continuous variables as median (IQR). The p-values are calculated using ^#^Fisher’s exact test, ^$^Mann-Whitney U test, and the chi-square test. *p-value significant at p ≤ 0.05, **p-value significant at p ≤ 0.01 IQR: Interquartile range, CVD: Cardiovascular disease

Variables		CVD risk			Chi-square	
		Low; n (%)	Mild/moderate; n (%)	Total; n (%)		
					z statistics	p-value
Addictions, diet, exercise						
Smoking/tobacco					13.785	<0.0001**
No		62 (61.4%)	39 (38.6%)	101 (80.2%)		
Yes		5 (20.0%)	20 (80.0%)	25 (19.8%)		
Duration (years), median (IQR)^$^		10 (6, 10)	20 (13.5, 27.5)	20 (10, 25)	-1.908	0.056
Alcohol consumption					10.247	0.001**
No		60 (60.6%)	39 (39.4%)	99 (78.6%)		
Yes		7 (25.9%)	20 (74.1%)	27 (21.4%)		
Duration (years), median (IQR)^$^		15 (10, 20)	16 (7.5, 27.5)	15 (8, 22)	-0.501	0.616
Addictions (smoking/tobacco/alcohol)					14.383	<0.0001**
No	57 (64.0%)		32 (36.0%)	89 (70.6%)		
Yes	10 (27.0%)		27 (73.0%)	37 (29.4%)		
Non-vegetarian consumption					2.075	0.150
No	16 (43.2%)		21 (56.8%)	37 (29.4%)		
Yes	51 (57.3%)		38 (42.7%)	89 (70.6%)		
Fruits consumption					0.790	0.622^#^
No	3 (75.0%)		1 (25.0%)	4 (3.2%)		
Yes	64 (52.5%)		58 (47.5%)	122 (96.8%)		
Vegetable consumption						
No	0 (0.0%)		0 (0.0%)	0 (0.0%)		
Yes	67 (53.2%)		59 (46.8%)	126 (100.0%)		
Additional salt consumption					0.407	0.523
No	59 (52.2%)		54 (47.8%)	113 (89.7%)		
Yes	8 (61.5%)		5 (38.5%)	13 (10.3%)		
Bakery products consumption					0.567	0.452
No	24 (49%)		25 (51%)	49 (38.9%)		
Yes	43 (55.8%)		34 (44.2%)	77 (61.1%)		
Daily time spent sitting (hours)					0.737	0.391
≤6 hours	49 (55.7%)		39 (44.3%)	88 (69.8%)		
>6 hours	18 (47.4%)		20 (52.6%)	38 (30.2%)		
Median (IQR)^$^	5 (3, 7)		6 (4, 8)	5 (3, 8)		0.556
Regular exercise					0.375	0.540
No	13 (59.1%)		9 (40.9%)	22 (17.5%)		
Yes	54 (51.9%)		50 (48.1%)	104 (82.5%)		
Duration of daily exercise (minutes)					0.840	0.847^#^
15 minutes	2 (66.7%)		1 (33.3%)	3 (2.9%)		
30 minutes	19 (48.7%)		20 (51.3%)	39 (37.5%)		
60 minutes	33 (53.2%)		29 (46.8%)	62 (59.6%)		
Median (IQR)	60 (30, 60)		60 (30, 60)	60 (30, 60)	-0.239	0.811
Total duration from starting exercising (years)					0.498	0.481
≤10 years	36 (54.5%)		30 (45.5%)	66 (63.5%)		
>10 years	18 (47.4%)		20 (52.6%)	38 (36.5%)		
Median (IQR)^$^	9 (4, 15)		8 (4, 20)	8 (4, 20)		0.674
Doing exercise on doctor's advice					3.343	0.068
No	45 (57.0%)		34 (43.0%)	79 (76.0%)		
Yes	9 (36.0%)		16 (64.0%)	25 (24.0%)		

**Table 3:** Association of health-related factors with CVD risk among university employees Descriptive statistics for categorical variables are shown as n (%) and for continuous variables as median (IQR). The p-values are calculated using ^#^Fisher’s exact test, ^$^Mann-Whitney U test, and the chi-square test. *p-value significant at p ≤ 0.05, **p-value significant at p ≤ 0.01 IQR: Interquartile range, CVD: Cardiovascular disease, NCD: Noncommunicable disease, BP: Blood pressure, SBP: Systolic blood pressure, DBP: Diastolic blood pressure, NCD: Non-communicable disease

Variables	CVD risk			Chi-square/	
	Low; n (%)	Mild/moderate; n (%)	Total; n (%)		
				z statistics	p-value
Health-related					
Pre-existing NCD				4.911	0.027*
No	52 (59.77%)	35 (40.23%)	87 (69.1%)		
Yes	15 (38.46%)	24 (61.54%)	39 (30.9%)		
Heart rate (bpm)				3.829	0.086^#^
Normal	63 (56.3%)	49 (43.8%)	112 (88.9%)		
Tachycardia	0 (0.0%)	0 (0.0%)	0 (0.0%)		
Bradycardia	4 (28.6%)	10 (71.4%)	14 (11.1%)		
Median (IQR)^$^	84 (76, 88)	86 (73, 93)	84 (75, 91)	-1.160	0.246
Body mass index (kg/m^2^)				5.279	0.151^#^
Normal	23 (51.1%)	22 (48.9%)	45 (35.7%)		
Overweight	39 (57.4%)	29 (42.6%)	68 (54%)		
Obese	3 (27.3%)	8 (72.7%)	11 (8.7%)		
Underweight	2 (100.0%)	0 (0.0%)	2 (1.6%)		
Median (IQR)^$^	25.7 (23.6, 27.6)	25.9 (23.4, 27.9)	25.7 (23.5, 27.6)	-0.337	0.736
Waist-to-hip ratio (WHR) status				1.821	0.402
Low	33 (47.8%)	36 (52.2%)	69 (54.8%)		
Moderate	18 (58.1%)	13 (41.9%)	31 (24.6%)		
High	16 (61.5%)	10 (38.5%)	26 (20.6%)		
Median (IQR)^$^	0.95 (0.93, 0.97)	0.95 (0.93, 0.97)	0.95 (0.93, 0.97)	-0.017	0.986
Perceived stress score categories				1.330	0.510^#^
Low	27 (57.4%)	20 (42.6%)	47 (37.3%)		
Moderate	38 (52.1%)	35 (47.9%)	73 (57.9%)		
High	2 (33.3%)	4 (66.7%)	6 (4.8%)		
Median (IQR)^$^	15 (11, 19)	18 (12, 22)	16 (12, 21)	-1.464	0.143
Blood pressure (mmHg)					
SBP Median (IQR)^$^	126 (118, 135)	143 (130, 157)	133 (122, 145)	-5.527	<0.0001**
DBP Median (IQR)^$^	80 (73, 87)	87 (79, 98)	84 (75, 91)	-3.821	<0.0001**
BP status				35.949	<0.0001**
Normal	38 (76%)	12 (24%)	50 (39.7%)		
High Normal	16 (69.6%)	7 (30.4%)	23 (18.3%)		
Grade I HT	13 (35.1%)	24 (64.9%)	37 (29.4%)		
Grade II HT	0 (0.0%)	16 (100.0%)	16 (12.7%)		
Respondent category				25.569	<0.0001**
Normal	42 (79.2%)	11 (20.8%)	53 (42.1%)		
Old Case of HT	15 (38.5%)	24 (61.5%)	39 (31.0%)		
New Case of HT	10 (29.4%)	24 (70.6%)	34 (27.0%)		

From univariate logistic regression analysis, it was observed that the odds of mild/moderate risk of CVDs was about two to three times significantly higher in male employees (OR=3.31, 95% CI=1.015-10.793, p=0.047), those working in academics (OR=2.27, 95% CI=1.096-4.683, p=0.027), those having a monthly income >Rs 30,000/- (OR=2.54, 95% CI=1.160-5.564, p=0.020), and having pre-existing NCDs (OR=2.38, 95% CI=1.096-5.157, p=0.028). More than five times higher odds of mild/moderate CVD risk were observed among permanent employees (OR=5.60, 95% CI=2.109-14.878, p=0.001) and those who reported addictions (OR=4.81, 95% CI=2.066-11.195, p<0.0001), employees who were already hypertensive at the time of study (OR=6.11, 95% CI=2.421-15.416, p<0.0001) and newly diagnosed during the study (OR=9.16, 95% CI=3.397-24.721, p<0.0001). In the multivariable model, one unit increase in age (adjusted odds ratio (AOR)=1.46, 95% CI=1.229-1.745, p<0.0001), BMI (AOR=1.25, 95% CI=1.001-1.553, p=0.049) and SBP (AOR=1.13, 95% CI=1.035-1.224, p=0.005) increasing the odds of mild/moderate CVD risk by 1.46, 1.25, and 1.13 times, respectively, among the university employees. The 6.49 times higher odds of mild/moderate risk were observed in regular employees (AOR=6.49, 95% CI=1.014-41.534, p=0.048) (Table 4).

**Table 4:** Univariate and multivariable logistic regression analysis of factors affecting CVD risk among university employees *p-value significant at p ≤ 0.05, **p-value significant at p ≤ 0.01 CVD: Cardiovascular diseas, NCD: Non-communicable diseas, SBP: Systolic blood pressure, DBP: Diastolic blood pressure, INR: Indian rupees

CVD risk - Mild/moderate (Ref: Low)	Univariate		Multivariable	
	OR (95% CI)	p-value	AOR (95% CI)	p-value
Sociodemographic				
Age (years)	1.27 (1.162-1.381)	<0.0001**	1.46 (1.229-1.745)	<0.0001**
Male (Ref: Female)	3.31 (1.015-10.793)	0.047*	2.02 (0.254-16.017)	0.507
Nuclear family (Ref: Joint)	1.93 (0.910-4.092)	0.087		
Total household members >4 (Ref: ≤4)	0.43 (0.210-0.898)	0.024*		
Relationship with family head, other (Ref: Self)	0.34 (0.148-0.795)	0.013*		
Graduate & above (Ref: Below graduation)	1.37 (0.578-3.245)	0.476		
Work-related				
Academic (Ref: Non-academic)	2.27 (1.096-4.683)	0.027*	1.78 (0.481-6.597)	0.388
Regular service (Ref: Contract)	5.60 (2.109-14.878)	0.001**	6.49 (1.014-41.534)	0.048*
Monthly income (INR) >30,000/-(Ref: ≤30,000/-)	2.54 (1.160-5.564)	0.020*		
Teaching (Ref: Non-teaching)	1.29 (0.591-2.820)	0.521		
Class 3 & 4 (Ref: Class 1 & 2)	0.70 (0.346-1.426)	0.328		
Service experience (years)	1.15 (1.089-1.213)	<0.0001**		
Nature of work (Ref: Professional)				
Office	0.66 (0.270-1.618)	0.364		
Skilled/other	0.87 (0.375-2.036)	0.755		
Addictions, diet, exercise				
Smoking/tobacco/alcohol (Ref: No addictions)	4.81 (2.066-11.195)	<0.0001**		
Non-vegetarian consumption (Ref: No)	0.57 (0.262-1.231)	0.152		
Fruit consumption (Ref: No)	2.72 (0.275-26.872)	0.392		
Additional salt consumption (Ref: No)	0.68 (0.211-2.215)	0.525		
Bakery products consumption (Ref: No)	0.76 (0.370-1.557)	0.452		
Daily sitting time (hrs)	1.04 (0.892-1.204)	0.640		
Regular exercise (Ref: No)	1.34 (0.526-3.400)	0.541		
Regular exercise duration (minutes)	1.00 (0.973-1.022)	0.823		
Health-related				
Pre-existing NCD (Ref: No)	2.38 (1.096-5.157)	0.028*	1.58 (0.447-5.585)	0.477
Heart rate (bpm)	1.02 (0.994-1.051)	0.127		
Bradycardia (Ref: Normal)	1.79 (0.975-3.297)	0.060		
Body mass index (kg/m2)	1.05 (0.959-1.158)	0.278	1.25 (1.001-1.553)	0.049*
Waist-to-hip ratio (Ref: Low)				
Moderate	0.66 (0.281-1.558)	0.345		
High	0.57 (0.228-1.438)	0.236		
Perceived stress score	1.04 (0.985-1.106)	0.150		
SBP (mmHg)	1.08 (1.046-1.113)	<0.0001**	1.13 (1.035-1.224)	0.005**
DBP (mmHg)	1.07 (1.032-1.107)	<0.0001**	1.00 (0.904-1.111)	0.962
Respondent category (Ref: Normal)				
Old Case of HT	6.11 (2.421-15.416)	<0.0001**		
New Case of HT	9.16 (3.397-24.721)	<0.0001**		

## Discussion

In our study, we found a considerably high prevalence of behavioural and lifestyle-related CVD risk factors, which are comparable with those found in the National Non-communicable Disease Monitoring Survey (NNMS), which was conducted in 2017-2018 and was the first systematic survey done across the Indian population to estimate risk factor prevalence in adults [28]. We observed clustering of ≥3 risk factors in more than three-fourths (n = 94, 74.6%) of university employees, which is significantly higher than in NNMS (40%). Similarly, we found a significantly higher prevalence of raised BP (n=53, 42%) compared to NNMS values (28.5%).

In addition, we found a higher prevalence of other risk factors as compared to NNMS, namely current use of alcohol (n=27 (21%); (NNMS: 15.9%)), overweight (n=68 (54%); (NNMS: 26%)), obesity (n=11(8.7%); (NNMS: 6%)), raised blood sugar levels (n=15 (12%); (NNMS: 9.3%). The main reason could be the variation in eligible employees (age ≥40 years) sample size (n=113) as compared to that of NNMS, which is n=10569, and included people between the age group of 18 to 69 years. Our study found that three-fourths (n=94, 74.6%) of the employees reported clustering of CVD risk factors(≥3), which is substantially higher than the rate reported in NNMS (40%). The 10-year risk of developing CVDs >10% in university employees was 17.5% (n=20), comparable with NNMS results (19%). Similar to the NNMS study, a recently published longitudinal ageing study across the Indian population (LASI) (n=11,621) has confirmed that 24.7% of the Indian population are at risk of developing CVDs in the next 10 years [29]. Although we found a relatively low proportion of employees (17.5%) were at 10-year-risk for developing CVDs as compared to both NNMS (19%) as well as LASI (24.7%), there are two possible reasons for this: one, the smaller sample size in our study, and two, the use of the WHO non-lab-based risk estimation charts in our study.

Among CVD risk factors, the current use of tobacco for smoking, harmful use of alcohol, lack of physical activity, and higher BMI are other substantial risk factors in university employees, were found to be on the higher side as reported in other studies done across India, but no significant association was seen between these risk factors and CVD risk except for smoking (OR=4.81, CI= 2.066-11.195, p<.0001) [10,22,23]. The prevalence of hypertension, recorded at 42.1% (n=126), was identified as the most significant CVD risk factor within our study cohort, which is markedly elevated in comparison to other recent investigations involving Indian university personnel conducted in Baroda (17%, n=1025). However, this figure is comparatively lower than that reported in another study conducted at Kakatiya University (Warangal, TG, IND), which documented a higher prevalence of 51.6% (n=576) than our findings [22,23]. This discrepancy is attributed to variations in sample size across these studies, including our research, which has a relatively smaller sample size compared to other studies, thereby underscoring the need for further investigations aimed at increasing the sample size.

In our investigation, we identified a noteworthy correlation between various non-modifiable risk factors, including advancing age, and modifiable risk factors, such as being overweight, obesity, and hypertension, all of which exhibit a significant association with the risk of CVD. Although a single study was conducted among university employees within an Indian context to evaluate CVD risk factors, two separate investigations analysing the risk factors associated with hypertension revealed that hypertension was the most prominent CVD risk factor among university employees [10,22,23]. Numerous studies conducted on a global scale in recent years have corroborated these similar conclusions [11-15]. Overall, university employees are found to have a significant number of CVD risk factors and are at a higher risk of developing CVDs in the future. Further studies with a larger sample size are needed to confirm these findings and develop an effective strategy for preventing CVDs among university employees.

Worksites represent an optimal environment for initiating CVD prevention and health promotion initiatives, contingent upon the active engagement of all organizational stakeholders, including employees, management, and governing authorities [19]. Health programs within workplace settings in higher education institutions are increasingly acknowledged as vital for fostering employee well-being and mitigating occupational stress. These initiatives aim to enhance health outcomes, improve work-life balance, and bolster employee retention rates. The execution of such programs exhibits significant variability worldwide, shaped by regional health priorities, cultural dynamics, and available resources [19-21]. Nevertheless, empirical studies remain limited concerning evaluating employee health and implementing workplace health promotion initiatives within Indian university contexts. No comprehensive research has been conducted across Indian universities regarding the establishment of worksite health promotion programs. The findings from our investigation highlight the urgent need to address the escalating prevalence of CVDs among university employees in India, alongside the development of a culturally sensitive and locally adaptable health promotion program tailored specifically for universities. This program aims to foster healthy lifestyles among university personnel and consequently alleviate the CVD burden within this demographic.

Limitations

The university where this study was carried out, being the sole institution of higher learning designated for a single district, exhibits a limited workforce in comparison to other state universities within the state of Maharashtra. Consequently, the results of this investigation cannot be extrapolated to analogous populations in Maharashtra or across India. Nevertheless, these results may be a foundation for conducting analogous research within alternative university environments, incorporating a larger participant pool.

A disproportionate male-to-female employee ratio within the examined population influences the study outcomes. To address this issue, we propose that subsequent researchers strive to establish a more equitable demographic representation to mitigate gender bias. Due to financial limitations, we utilised the non-laboratory-based CVD risk assessment charts provided by the WHO to evaluate the risk of CVD development among employees. This methodology constrained our ability to screen individuals younger than 40. We strongly advocate that future researchers utilize WHO laboratory-based assessment charts and conduct screenings of the entire employee cohort within a study population to ascertain CVD risk.

## Conclusions

Our study found that age increase, body mass index, and SBP were significant factors associated with the mild to moderate risk of CVDs among regular university employees. There is a high prevalence of CVD risk factors among university employees, especially hypertension, lack of physical activity, and habits such as smoking and harmful use of alcohol. These physiological and behavioural risk factors act synergistically, making university employees prone to developing CVDs in their future lives. There is an urgent need to conduct opportunistic screening of university employees in India for CVD risk factors. There is also a need to assess the occupational stress perceived by university employees. Findings from such research can be utilized to design an appropriate worksite CVD prevention program with a particular focus on behavioural and lifestyle-related changes.
